# Alterations in the nigrostriatal system following conditional inactivation of α-synuclein in neurons of adult and aging mice

**DOI:** 10.1016/j.neurobiolaging.2020.02.026

**Published:** 2020-07

**Authors:** Natalia Ninkina, Tatiana V. Tarasova, Kirill D. Chaprov, Andrei Yu Roman, Michail S. Kukharsky, Larisa G. Kolik, Ruslan Ovchinnikov, Aleksey A. Ustyugov, Andrey D. Durnev, Vladimir L. Buchman

**Affiliations:** a School of Biosciences, Cardiff University, Cardiff, United Kingdom; b Institute of Physiologically Active Compounds Russian Academy of Sciences (IPAC RAS), Moscow Region, Russian Federation; c FSBI Research Zakusov Institute of Pharmacology (FSBI RZIP), Moscow, Russian Federation; d Pirogov Russian National Research Medical University, Moscow, Russian Federation

**Keywords:** Dopaminergic neurons, Substantia nigra, Conditional knockout, Dopamine turnover, Parkinson's disease

## Abstract

The etiology and pathogenesis of Parkinson’s disease (PD) are tightly linked to the gain-of-function of α-synuclein. However, gradual accumulation of α-synuclein aggregates in dopaminergic neurons of substantia nigra pars compacta (SNpc) leads to the depletion of the functional pool of soluble α-synuclein, and therefore, creates loss-of-function conditions, particularly in presynaptic terminals of these neurons. Studies of how this late-onset depletion of a protein involved in many important steps of neurotransmission contributes to PD progression and particularly, to worsening the nigrostriatal pathology at late stages of the disease are limited and obtained data, are controversial. Recently, we produced a mouse line for conditional knockout of the gene encoding α-synuclein, and here we used its tamoxifen-inducible pan-neuronal inactivation to study consequences of the adult-onset (from the age of 6 months) and late-onset (from the age of 12 months) α-synuclein depletion to the nigrostriatal system. No significant changes of animal balance/coordination, the number of dopaminergic neurons in the SNpc and the content of dopamine and its metabolites in the striatum were observed after adult-onset α-synuclein depletion, but in aging (18-month-old) late-onset depleted mice we found a significant reduction of major dopamine metabolites without changes to the content of dopamine itself. Our data suggest that this might be caused, at least partially, by reduced expression of aldehyde dehydrogenase ALDH1a1 and could lead to the accumulation of toxic intermediates of dopamine catabolism. By extrapolating our findings to a potential clinical situation, we suggest that therapeutic downregulation of α-synuclein expression in PD patients is a generally safe option as it should not cause adverse side effects on the functionality of their nigrostriatal system. However, if started in aged patients, this type of therapy might trigger slight functional changes of the nigrostriatal system with potentially unwanted additive effect to already existing pathology.

## Highlights

• Efficient depletion of striatal α-synuclein after adult-onset inactivation of *Snca*.
• Nigrostriatal system is not markedly affected by adult-onset depletion of α-synuclein.
• Changes to striatal dopamine metabolism after α-synuclein depletion in aging animals.
• Downregulation of ALDH1a1 as a possible cause of striatal dopamine metabolism changes.

## 1 Introduction

For over two decades, since the discovery of the first missense mutation in the gene encoding α-synuclein as the cause of a familial form of Parkinson’s disease (PD) (Polymeropoulos et al., 1997) and identification of α-synuclein as a major component of Lewy bodies and Lewy neurites (Spillantini et al., 1997, Spillantini et al., 1998), malfunction of this protein is considered as a key molecular event in the pathogenesis of PD and certain other diseases collectively known as α-synucleinopathies (Galvin et al., 2001, Goedert, 1999, Spillantini and Goedert, 2000). The prevalent hypothesis supported by multiple lines of experimental and clinical evidence proposes the gain-of-function mechanism associated with the formation of toxic α-synuclein species as the cause of pathological changes in affected cells of the nervous system (reviewed in (Caughey and Lansbury, 2003, Dev et al., 2003, Fink, 2006, Uversky, 2007, Uversky, 2017)). Nevertheless, progressive accumulation of final products of α-synuclein aggregation in pathological inclusions might lead to a gradual depletion of a pool of functional protein. Indeed, recent in vivo studies of neurons expressing GFP-fused α-synuclein demonstrated reciprocal maturation of α-synuclein inclusions and decrease of soluble α-synuclein in the same cell (Osterberg et al., 2015). In neurons, including dopaminergic neurons of substantia nigra pars compacta (SNpc), whose dysfunction is responsible for most severe symptoms of PD, α-synuclein is normally located in presynaptic terminals where it modulates various molecular processes involved in synthesis, storage, release, and reuptake of neurotransmitters (Abeliovich et al., 2000, Burre et al., 2010, Cabin et al., 2002, Chandra et al., 2005, Fountaine and Wade-Martins, 2007, Greten-Harrison et al., 2010, Lee et al., 2001, Logan et al., 2017, Nakamura et al., 2011, Nemani et al., 2010, Perez et al., 2002, Vargas et al., 2017, Wersinger and Sidhu, 2003, Westphal and Chandra, 2013). Therefore, the deficiency of functional α-synuclein might compromise the efficiency of synaptic transmission, and thus, contributes to the nigrostriatal system disfunction in PD patients (Al-Wandi et al., 2010, Benskey et al., 2016, Collier et al., 2016, Venda et al., 2010).

However, modeling of α-synuclein deficiency in mice by constitutively knocking out the encoding gene did not support this hypothesis because the complete loss of α-synuclein in these animals did not have a substantial effect on synaptic transmission in general and dopamine neurotransmission in particular (discussed in a recent review (Sulzer and Edwards, 2019)). Still, modest impairments of the nigrostriatal system function were revealed in aged, i.e. 2-year-old α-synuclein deficient mice (Al-Wandi et al., 2010, Connor-Robson et al., 2016). A feasible explanation is that during a period of high plasticity, neurons of the developing nervous system get adapted to the absence of α-synuclein. Lost α-synuclein function can be compensated by other members of the synuclein family, β-synuclein and/or γ-synuclein, but even in their absence in triple synuclein null mutant mice, an efficient compensation can be achieved by certain unknown mechanism(s). This functional compensation might last for a long time with some defects become evident only in aged triple synuclein null mutant animals (Anwar et al., 2011, Burre et al., 2010, Connor-Robson et al., 2016, Greten-Harrison et al., 2010).

The depletion of neurons from α-synuclein in adult or aging animals would leapfrog mechanisms of developmental compensation that make neurons resistant to potentially negative effects of α-synuclein deficiency and should better represent processes that might take place in the nervous system of PD patients. The recent high interest in the reduction of α-synuclein level as a therapeutic approach to prevent PD progression makes it more than ever important to obtain strong experimental evidence that α-synuclein loss-of-function induced in neurons of the adult or aging nigrostriatal system does not cause adverse effects in the long term. However, previous studies of the nigrostriatal system in mammals with partial depletion of α-synuclein following stereotaxic injection into the substantia nigra region of adult animals of specific siRNA or viruses encoding specific shRNA produced inconsistent results, and in some cases, directly opposite results (Collier et al., 2016, Cooper et al., 2014, Gorbatyuk et al., 2010, Lewis et al., 2008, McCormack et al., 2010, Zharikov et al., 2015).

Recently, we produced a line of mice with genetic modification that allows efficient conditional inactivation of an α-synuclein-encoding gene (Ninkina et al., 2015), and here, we employed this model to study the long-term consequences to the nigrostriatal system of complete α-synuclein depletion from neurons of adult and aging mice.

## 2 Materials and methods

### 2.1 Production of experimental mouse cohorts and induction of Cre-recombination

All parental mouse lines used in this study were transferred to C57Bl6J (Charles River) genetic background by multiple (>10) generations of backcrosses. Animals were maintained in conventional open-lid cages with *ad libitum* access to standard chow and water. For producing animal cohorts for conditional inactivation of α-synuclein-encoding *Snca* gene and relevant control animals, mice homozygous for loxP-flanked second exon of the *Snca* gene with removed neo-cassette (*Snca* ^*floxΔneo/floxΔneo*^ (Ninkina et al., 2015)) were crossed with mice heterozygous for constitutively inactivated *Snca* gene (Abeliovich et al., 2000) and homozygous for a transgenic cassette for expression of Cre-ERT2 recombinase under control of a neuro-specific enolase (NSE) promoter (obtained from Jean C. Manson, University of Edinburgh). Thus, all animals produced by this cross expressed Cre-ERT2 recombinase in their neurons and carried one allele of the *Snca* gene with loxP-flanked second exon (*Snca* ^*flox*^), while the second allele of the gene was either fully functional (*Snca*^*+*^; α^*+*^) or constitutively inactivated (*Snca*^*-*^; α^*-*^). At the age of 6 months, *Snca* ^*flox/-*^ mice of the same sex from the same litter were distributed, in equal numbers, wherever possible, into an experimental group that received tamoxifen injections and a control group that received vehicle injection. Each of these two groups contained three cohorts of at least 12 males and 12 females for behavioral, histological, and biochemical studies at the age of 10, 14, and 18 months. The third group of *Snca* ^*flox/-*^ mice was left aging and received tamoxifen injections at the age of 12 months; these animals were tested, and their brain tissues collected at the age of 18 months along with the last cohort of mice injected at the age of 6 months. Inactivation of *Snca* ^*flox*^ gene by loxP recombination was achieved following activation of Cre-ERT2 recombinase by 5 days of i.p. injection of tamoxifen (0.5 mmol/kg dissolved in corn oil). Because for all studied parameters, similar results were obtained for male and female groups, combined data for both genders are shown if not stated otherwise.

All animal work was carried out in accordance with the United Kingdom (Scientific Procedures) Act (1986) and European Directive EC 86/609, and has been approved by the Cardiff University Ethical Review Committee and the Home Office (Project Licences 30/2844 and 30/3412).

### 2.2 Genotyping

Animal genotypes were determined by PCR analysis of DNA from ear biopsies collected as a part of the identification process. Genotyping for *Snca* gene variants was carried out as described previously (Abeliovich et al., 2000, Ninkina et al., 2015, Roman et al., 2017). To detect the presence of the Cre-ERT2 expression cassette in the mouse genome and discriminate between hemizygous and homozygous animals, a real-time quantitative PCR (primers: 5′-ATACCGGAGATCATGCAAGC-3′ and 5′- CCTGTTTCACTATCCAGGTTACG-3) and backcross analysis were used, as described elsewhere (Ninkina et al., 2009).

### 2.3 Behavioral tests

Inverted grid and accelerated rotarod tests were carried as described previously (Connor-Robson et al., 2016, Robertson et al., 2004). Locomotion activity of mice in a novel environment was assessed in an activity camera (UgoBasile) according to previously published protocols (Anwar et al., 2011). Animals were not tested repeatedly in behavioral tests, i.e. cohorts of animals designated for testing at the age of 18 months were not used for testing at the age of 10 or 14 months.

### 2.4 Preparation of histological sections, immunohistochemistry, and neuronal cell counts

Mice were terminated by a Schedule 1 method, and brains were dissected. Cerebral cortices were carefully unfolded to expose striata; a dorsal part of each striatum was pinched out using a curved forceps, and after cortices were folded back, the brains were fixed and proceeded for histology. For some animals dissected brains were cut sagitally with a blade, and one hemisphere was used for dissecting the midbrain region for RNA or protein extraction, whereas another hemisphere was fixed for histology. Fixation, preparation of histological sections, staining with antibody against tyrosine hydroxylase (TH, mouse monoclonal antibody, clone TH-2, Sigma diluted 1:1000) and stereological counting of TH-positive neurons in the SNpc and ventral tegmental area (VTA) were performed as described (Al-Wandi et al., 2010, Connor-Robson et al., 2016, Robertson et al., 2004). Briefly, the borders of the substantia nigra and ventral tegmental area (VTA) on stained sections were outlined using distribution atlas of TH-positive cells (Hokfelt et al., 1984). The first section for counting was randomly chosen from the first ten sections that included the SNpc/VTA region. Starting from this section, on every fifth section, TH-positive cells with a clearly visible nucleus were counted through the whole region. The Axiovision imaging program (Carl Zeiss Vision) was employed to measure diameters of 30 nuclei of dopaminergic neurons in each of these regions of every mouse brain included in this study. The nuclei were chosen randomly, and the distance measured as the horizontal length as they appeared on the screen. A mean was calculated for each animal and used for Abercrombie’s correction (Abercrombie, 1946) to obtain an actual number of TH positive cells in the structure.

### 2.5 Analysis of striatal neurochemicals by high-pressure liquid chromatography (HPLC)

Biopsies of dorsal striata were snap-frozen in liquid nitrogen and kept at −80 °C. Concentrations of striatal dopamine, 3,4-dihydroxyphenylacetic acid (DOPAC) and homovanillic acid (HVA) in cleared extracts obtained by homogenization of each biopsy in 300 μL of 0.06 M HClO_4_ followed by centrifugation at 15000*g* for 5 minutes at 4 °C were measured by HPLC as described previously (Connor-Robson et al., 2016).

### 2.6 Analysis of mRNA expression

Total RNA was extracted from dissected midbrains of 18-month-old mice injected with tamoxifen at the age of 12 months and control littermates using the RNeasy Plus Mini kit (Qiagen). Analysis of RNA expression using a custom nCounter CodeSet was carried out in the nanoSTRING nCounter Core Facility of the University College London. The first-strand cDNA for real-time quantitative RT-PCR analysis was synthesized using random primers (Promega) and SuperScriptIII reverse transcriptase (Invitrogen). For PCR amplification reaction Go Taq Hot Start polymerase (Promega), DyNAmo HS SYBR Green supermix, and ROX (Finnzymes) as a passive reference dye, were used. Each sample was analyzed in quadruplicate on an ABI StepOnePlus real-time PCR instrument, and data were analyzed using integrated StepOne Software v2.3 (Applied Biosystems). cDNA amount for each gene was normalized to that of GAPDH. Primer sequences used were as follows: ALDH1a1: 5′- GGCCTTCACTGGATCAACAC-3′ and 5′- GGGTGACTCTCTTCAGATTG-3’; GAPDH: 5′- TCGCCAGCCGAGCCA-3′ and 5′- GAGTTAAAAGCAGCCCTGGTG -3’.

### 2.7 Western blotting

Total protein samples were prepared by homogenization of mouse striata in the SDS-PAGE loading buffer following incubation for 10 minutes at 100 °C. Gel electrophoresis, transfer to PVDF membrane, incubation with primary and secondary HRP- or Cy5-conjugated antibodies (GE Healthcare and Molecular Probes, correspondingly), and detection of protein bands was performed as described previously (Ninkina et al., 2003, Ninkina et al., 2012). Primary antibodies against α-synuclein (mouse monoclonal antibody, clone 42, BD Transduction Laboratories, diluted 1:1000), β-synuclein (rabbit monoclonal, clone EP1537Y, Abcam, diluted 1:5000), TH (mouse monoclonal, clone TH-2, Sigma, diluted 1:5000), synaptophysin (mouse monoclonal, clone 2, BD Transduction Laboratories, diluted 1:10,000), aldehyde dehydrogenase ALDH1a1 (rabbit polyclonal ABD12, Merck, diluted 1:4000) and β-actin (mouse monoclonal, clone AC-15, Sigma, diluted 1:10,000) were used.

### 2.8 Statistical analysis

All data are presented as means ± SEM. ANOVA, paired *t*-test, or nonparametric Mann-Whitney test was used for assessing the statistical significance of the difference between groups, as appropriate. The nSolver 4.0 software (NanoString Technologies) was used for analyzing mRNA expression data obtained by NanoString/nCounter technique. Statistical analysis was performed using GraphPad Prism 7.0e.

## 3 Results

### 3.1 Depletion of α-synuclein in the dorsal striatum following activation of Cre-ERT2 recombinase in neurons of adult Snca ^flox/-^ mice

**Fig. 1:**
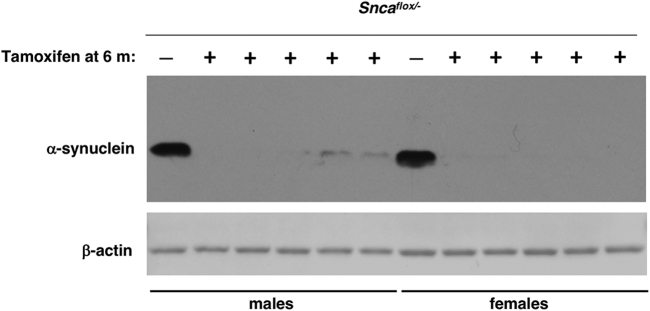
Depletion of α-synuclein in the striatum following conditional inactivation of the encoding gene. Western blot analysis of α-synuclein in the total protein samples of dissected dorsal striata of 10-month-old *Snca*^*flox/-*^ mice carrying a copy of NSE/Cre-ERT2 transgenic cassette following tamoxifen or vehicle injection at the age of 6 months. Individual mouse striata were analyzed. For loading control, the same blot was re-probed with an antibody against β-actin.

At the age of 6 months, heterozygous *Snca*^*flox/-*^ mice carrying a copy of NSE/Cre-ERT2 transgenic cassette received tamoxifen or vehicle injections as described in the Methods section. Four months later (i.e., at the age of 10 months), several male and female animals were sacrificed, dorsal striata were dissected, and expression of α-synuclein in total protein extracts was analyzed by Western blotting with an antibody specific to mouse α-synuclein. As illustrated in Fig. 1, in contrast to a high level in the vehicle-injected mice, α-synuclein becomes undetectable in the dorsal striatum of the majority of tamoxifen-injected mice with only trace amount remains in this brain structure of some animals, which might be caused by the presence of erythrocyte-derived α-synuclein originated from blood in the latter samples. A similar pattern was observed when the expression of α-synuclein was assessed in the striatum of 18-month-old mice that received tamoxifen at the age of 6 months (see below).

**Fig. 2:**
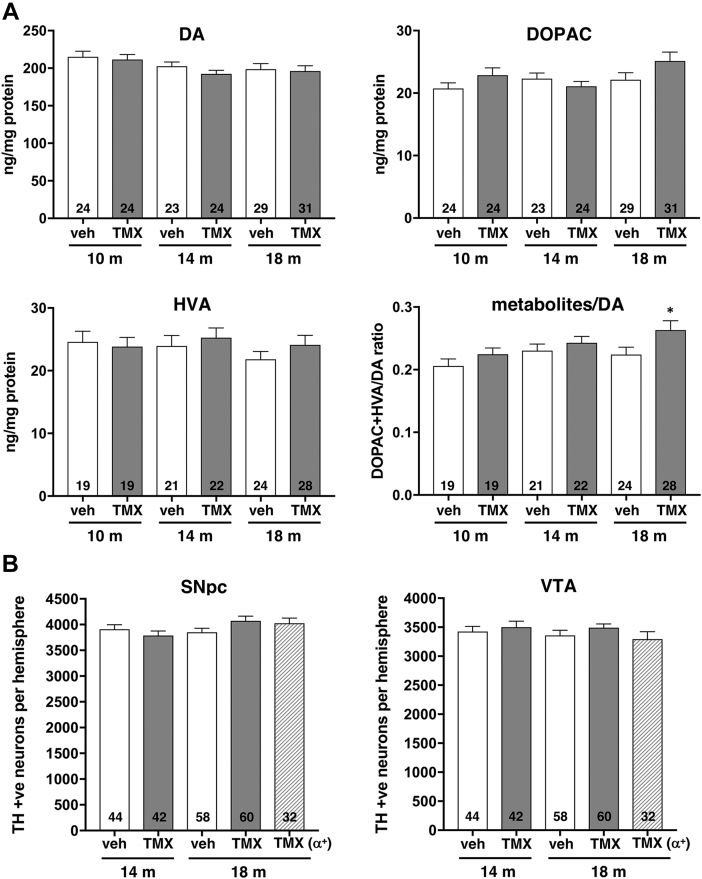
Striatal dopamine metabolism and populations of midbrain dopaminergic neurons are not affected by conditional inactivation of *Snca* gene in neurons of 6-month-old mice. (A) Dopamine (DA), 3,4-dihydroxyphenylacetic acid (DOPAC) and homovanillic acid (HVA) content and the ratio of metabolites (DOPAC+HVA) to dopamine in the dorsal striatum of 10-, 14- and 18-month-old *Snca*^*flox/-*^ mice carrying a copy of NSE/Cre-ERT2 transgenic cassette injected with tamoxifen (TMX) or vehicle (veh) at the age of 6 months. Bar charts show mean±SEM of neurochemical concentrations measured by HPLC with electrochemical detection or of their ratio. The number of samples analyzed for each group is shown at the bottom of the corresponding bar. Statistical analysis using one-way ANOVA revealed no statistically significant difference between groups for dopamine (F (5, 149) = 1.669; *p* = 0.1455), DOPAC (F (5, 149) = 2.125; *p* = 0.0655), HVA (F (5, 127) = 0.5943; *p* = 0.7043) but statistically significant increase in metabolites to dopamine ratio (F (5, 127) = 2.723; *p* = 0.0226) was observed. The *post-hoc* Sidak’s multiple comparisons test showed significant increase of this ratio in tamoxifen-injected compared to vehicle-injected mice at the age of 18 months (∗*p* = 0.0473). (B) The number of TH-positive neurons in the substantia nigra pars compacta (SNpc) and ventral tegmental area (VTA) of 14- and 18-month-old *Snca*^*flox/-*^ mice injected with tamoxifen (TMX) or vehicle (veh) at the age of 6 months. Results for a control group of *Snca*^*flox/+*^ mice (α^*+*^) injected with tamoxifen at the age of 6 months and analyzed at the age of 18 months are also included. Bar charts show mean±SEM of stereologically counted neuron numbers in each structure. The number of samples analyzed for each group is shown at the bottom of the corresponding bar. Statistical analysis using one-way ANOVA revealed no statistically significant difference between groups (F (4, 231) = 1.857; *p* = 0.1189 for SNpc and F (4, 231) = 0.8610; *p* = 0.4881 for VTA).

**Fig. 3:**
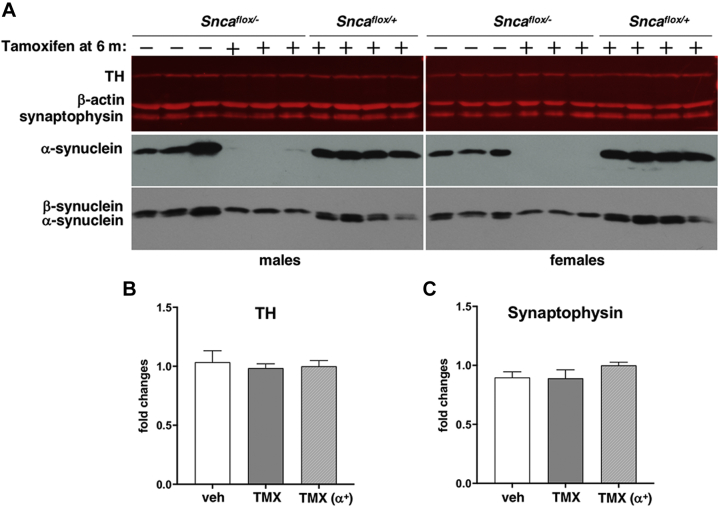
Expression of presynaptic proteins in the dorsal striatum of 18-month-old *Snca*^*flox/-*^ and *Snca*^*flox/+*^ mice injected with tamoxifen and *Snca*^*flox/-*^ mice injected with vehicle at the age of 6 months. (A) The upper panel shows a representative Western blot image illustrating the expression of tyrosine hydroxylase (TH) and synaptophysin with β-actin as a loading control. Individual mouse striata were analyzed. The Cy5-conjugated secondary antibody was used for the detection of these proteins. A bottom part of the same membrane was probed with an antibody against mouse α-synuclein (middle panel), and then, without stripping, was re-probed with an antibody against β-synuclein (bottom panel). HRP- conjugated secondary antibody and chemiluminescent detection were used for the detection of both synucleins. For quantification, protein bands were scanned, levels of TH (B) and synaptophysin (C) were normalized to levels of β-actin in studied striatal samples and then to the average level in control *Snca*^*flox/+*^ mice. Bar charts show means±SEM of normalized values obtained by Western blot analysis of 6 vehicle-injected (veh) and 6 tamoxifen-injected (TMX) *Snca*^*flox/-*^ mice and 4 tamoxifen-injected *Snca*^*flox/+*^ (TMX (α^+^)) mice. Statistical analysis using the Kruskal-Wallis test revealed no statistically significant difference between groups (*p* = 0.9704; Kruskal-Wallis statistic 0.06548 for TH and *p* = 0.2438; Kruskal-Wallis statistic 2.890 for synaptophysin).

### 3.2 Inactivation of an α-synuclein gene at the age of 6 months does not affect striatal content of dopamine and its metabolites

The contents of dopamine, DOPAC and HVA in the dorsal striatum of 10-, 14- and 18-month-old mice that received tamoxifen or vehicle at the age of 6 months were analyzed by HPLC with electrochemical detection. Statistically, no significant changes were observed between tamoxifen-injected and vehicle-injected animals for each of these neurochemicals at any of the studied age points (Fig. 2A). Although a trend for increased metabolites to dopamine ratio in tamoxifen-injected mice was noted at each age point, the difference reaches significance only for the largest studied groups of 18-month-old animals (*p* = 0.0473, one-way ANOVA with *post-hoc* Sidak’s multiple comparisons test; Fig. 2A).

### 3.3 No changes in the number of TH-positive neurons in the SNpc and VTA, TH expression in the striatum and performance in the balance and coordination tests of mice following inactivation of an α-synuclein gene at the age of 6 months

The number of dopaminergic neurons in two midbrain ganglia, SNpc and VTA, was assessed in *Snca*^*flox/-*^ and *Snca*^*flox/+*^ mice carrying a copy of NSE/Cre-ERT2 transgenic cassette eight and twelve months after tamoxifen or vesicle injections, i.e. at the age of 14 and 18 months. Stereological counting of TH-positive neurons on immunostained histological sections revealed no difference between studied groups in both ganglia (Fig. 2B, Suppl. Fig. A1).

Consistently with the lack of changes in the number of TH-positive neurons in the SNpc and dopamine content in the dorsal striatum, no difference in TH expression in the dorsal striatum of 18-month-old mice was detected by Western blot analysis between mice injected with tamoxifen and vehicle (Fig. 3). The expression of synaptophysin and β-synuclein in this brain structure was also found unaffected by α-synuclein depletion.

**Fig. 4:**
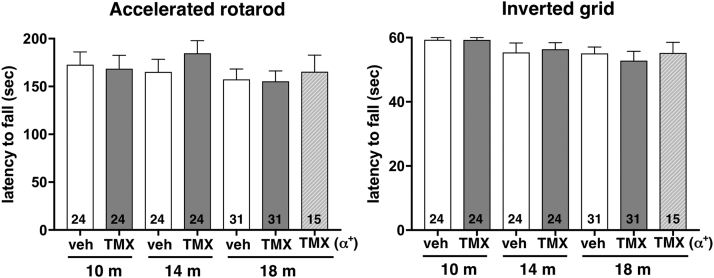
Assessment of balance and coordination of mice following conditional inactivation of *Snca* gene in their neurons at the age of 6 months. Accelerated rotarod and inverted grid performance of 10-, 14- and 18-month-old *Snca*^*flox/-*^ mice injected with tamoxifen (TMX) or vehicle (veh) at the age of 6 months. Results for 18-month-old tamoxifen-injected *Snca*^*flox/+*^ mice (TMX (α^*+*^)) are also shown. The number of assessed animals in each group is shown at the bottom of the corresponding bar. Statistical analysis using one-way ANOVA revealed no statistically significant difference between groups (F (6, 167) = 0.6728; *p* = 0.6718 for rotarod test and F (6, 166) = 1.138; *p* = 0.3426 for inverted grid test).

Not surprisingly, in the absence of biochemical and morphological changes in the nigrostriatal system, inactivation of an α-synuclein-encoding gene at the age of 6 months did not affect animal performance in the accelerated rotarod and inverted grid tests (Fig. 4).

### 3.4 Inactivation of α-synuclein gene at the age of 12 months does not affect the number of TH-positive neurons in the SNpc, dopamine content in the dorsal striatum of 18-month-old mice and their balance and coordination

For testing whether triggering of alpha-synuclein depletion in neurons of older animals would cause changes in their nigrostriatal system, a group of animals from the same cohort of *Snca*^*flox/-*^ mice was injected with tamoxifen at the age of 12 months and assessed at the age of 18 months.

**Fig. 5:**
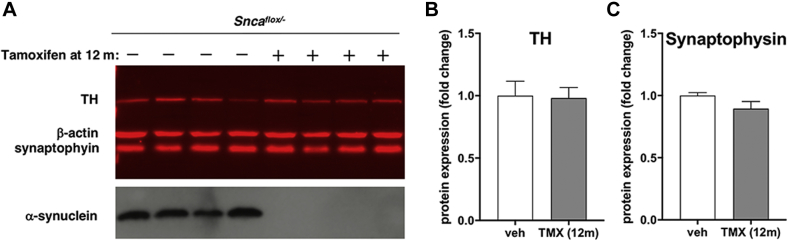
Expression of presynaptic proteins in the dorsal striatum of 18-month-old *Snca*^*flox/-*^ mice injected with tamoxifen or vehicle at the age of 12 months. (A) The upper panel shows a representative Western blot image illustrating the expression of tyrosine hydroxylase (TH) and synaptophysin with β-actin as a loading control. Individual mouse striata were analyzed. The Cy5-conjugated secondary antibody was used for the detection of these proteins. A bottom part of the same membrane was probed with an antibody against mouse α-synuclein and HRP- conjugated secondary antibody following chemiluminescent detection. For quantification, protein bands were scanned, levels of TH (B) and synaptophysin (C) in each striatal sample were normalized to the level of β-actin in this sample and then to the average level in control vehicle-injected mice. Bar charts show means±SEM of normalized values obtained by Western blot analysis of 6 vehicle-injected (veh) and 8 tamoxifen-injected (TMX) *Snca*^*flox/-*^ mice. Statistical analysis using the Mann-Whitney test revealed no statistically significant difference between groups (*p* = 0.5080; sum of ranks (137, 163); Mann-Whitney U = 58 for TH and *p* = 0.2284; sum of ranks (55, 50); Mann-Whitney U = 14 for synaptophysin).

**Fig. 6:**
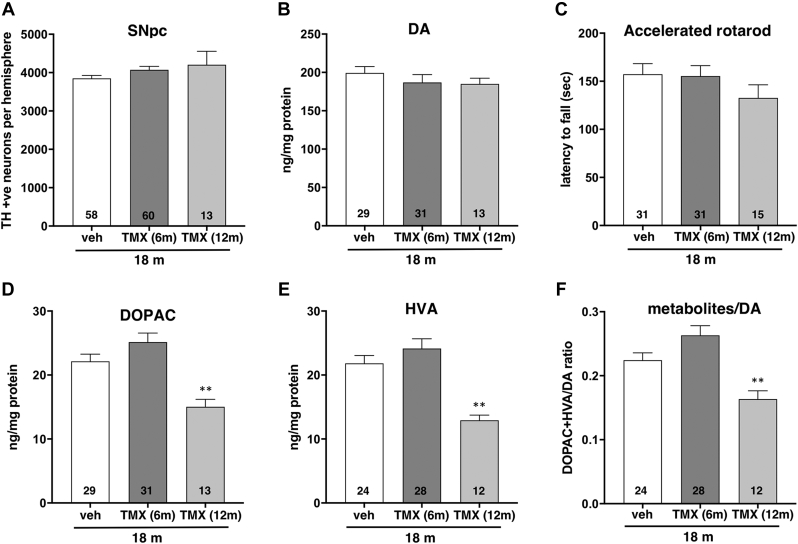
No changes in SNpc dopaminergic neuron complement, striatal dopamine content and rotarod performance but significant decrease of striatal dopamine metabolites in the striatum of 18-month-old mice following conditional inactivation of *Snca* gene in their neurons at the age of 12 months. Bar charts show mean±SEM of the number of TH-positive neurons in the SNpc (A), striatal dopamine (B), DOPAC (D), HVA (E) contents, DOPAC+HVA to dopamine ratio (F) and the latency to fall from an accelerated rotatod (C) for 18-month-old *Snca*^*flox/-*^ mice injected with vehicle (veh) and tamoxifen (TMX) at the age of either 6 or 12 months. The number of samples/animals analyzed for each group is shown at the bottom of the corresponding bar. Statistical analysis using one-way ANOVA revealed no difference between groups in neuron number (F (2, 128) = 2.011; *p* = 0.1380), rotarod performance (F (2, 74) = 0.9736; *p* = 0.3825) and dopamine content (F (2, 70) = 0.6066; *p* = 0.5480) but significant difference between groups in DOPAC content (F (2, 70) = 10.50; ∗∗*p* = 0.0001), HVA content (F (2, 61) = 12.02; ∗∗*p* <0.0001) and metabolites to dopamine ratio (F (2, 61) = 11.99; ∗∗*p* <0.0001). Animals injected with tamoxifen at the age of 12 months showed decrease in DOPAC content (*p* = 0.0064), HVA content (*p* = 0.0011) and the ratio (*p* = 0.0096) compared to animals injected with vehicle only and in DOPAC content (*p* <0.0001), HVA content (*p* <0.0001) and the ratio (*p* <0.0001) compared to animals injected with tamoxifen at the age of 6 months (all by Sidak’s multiple comparisons test).

Similar to the inactivation of the gene at the age of 6 months, this protocol led to efficient depletion of striatal α-synuclein and caused no changes to striatal TH and synaptophysin levels (Fig. 5). Consistently, no changes in TH-positive neuron number in the SNpc (Fig. 6A, Suppl. Fig. A1) and striatal dopamine context (Fig. 6B) were found in 18-month-old *Snca*^*flox/-*^ mice after inactivation of an α-synuclein-encoding gene at the age of 12 months when compared with vehicle-injected *Snca*^*flox/-*^ mice or *Snca*^*flox/-*^ mice injected with tamoxifen at the age of 6 months.^1^ The performance in the accelerated rotarod test was not significantly affected (Fig. 6C), and all animals in the group injected with tamoxifen at 12 months successfully completed the inverted grid test, suggesting that their balance and coordination are not compromised.

### 3.5 Changes in dopamine catabolism in the dorsal striatum of 18-month-old mice following inactivation of an α-synuclein gene at the age of 12 months

In spite of a normal content of dopamine, its catabolism in the dorsal striatum of 18-month-old mice becomes affected as the result of α-synuclein depletion from the age of 12 months, as evident from significantly reduced content of both major dopamine metabolites, DOPAC and HVA (Fig. 6D and E). This causes a substantial decrease in the metabolite to dopamine ratio (Fig. 6F).

**Fig. 7:**
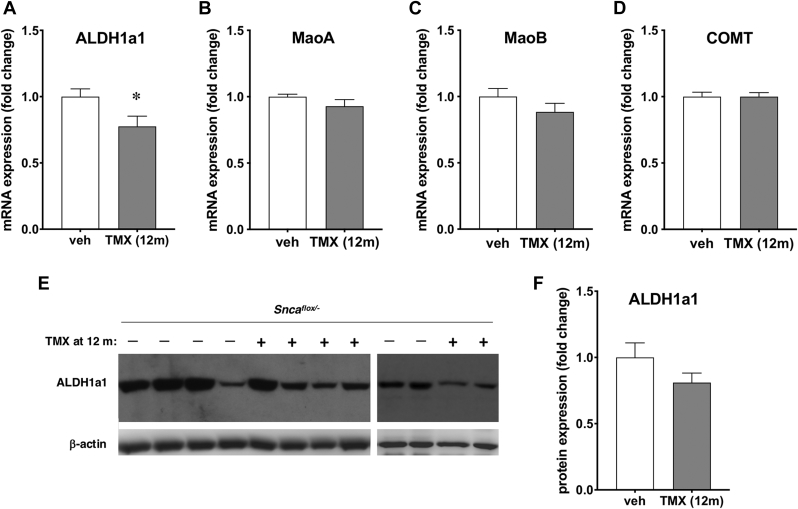
Decreased expression of aldehyde dehydrogenase ALDH1a1 in 18-month-old mice following conditional inactivation of *Snca* gene in their neurons at the age of 12 months. (A) Fold changes (mean ± SEM) of the expression of aldehyde dehydrogenase 1a1 (ALDH1a1) mRNA in the midbrain of tamoxifen-injected (TMX (12 m)) versus vehicle-injected (veh) mice assessed by real-time qRT-PCR. RNAs extracted from midbrains of 6 animals per group were analyzed in two technical repeats (∗*p* = 0.0167; sum of ranks (191, 109); Mann-Whitney U = 31). (B-D) Direct measurement of mRNA content by nanoString/nCounter technique revealed no significant expression differences (*p* >0.05, Mann-Whitney test) for mRNAs encoding catechol-O-methyl transferase (COMT), monoamine oxidase A (MaoA) and monoamine oxidase B (MaoB). (E, F) Images of two representative Western blots illustrating the expression of ALDH1a1 in the dorsal striatum with β-actin as a loading control. For quantification, protein bands were scanned, levels of ALDH1a1 were normalized to levels of β-actin in studied striatal samples and then to the average level in control vehicle-injected mice. Bar chart show means±SEM of normalized values obtained by Western blot analysis of 6 vehicle-injected and 10 tamoxifen-injected *Snca*^*flox/-*^ mice (*p* = 0.1471; sum of ranks (65, 71); Mann-Whitney U = 16).

A crucial final step in DOPAC and HVA production is an oxidation reaction that in dopaminergic neurons is primarily catalyzed by cytosolic aldehyde dehydrogenase ALDH1a1. The deficiency of this enzyme leads to the accumulation of dopamine catabolism intermediates, reactive aldehydes, DOPAL and HVAldehyde that are toxic to neurons. A significant downregulation of ALDH1a1 mRNA expression in the midbrain region of 18-month-old animals injected with tamoxifen at the age of 12 months when compared to control littermates injected with a vehicle was detected by quantitative RT-PCR (Fig. 7A). This downregulation in a region where cell bodies of SNpc dopaminergic neurons are located is consistent with a trend for reduction of the protein level in the dorsal striatum, although the difference revealed by quantitation of Western blot data from available samples did not reach statistical significance mainly because of inconsistent low ALDH1a1 level in one of the control samples (Fig. 7E and F). In contrast, no changes in the expression of mRNA encoding enzymes involved in the production of reactive aldehydes from dopamine, catechol-O-methyl transferase (COMT) and monoamine oxidases (MAO) were found (Fig. 7B–D). The latter results were obtained by NanoString/nCounter analysis of a bespoke codeset of mRNAs encoding proteins involved in neurotransmission. In this analysis, neither of 106 studied mRNAs displayed statistically significant fold change of expression in tamoxifen-injected versus control, vehicle-injected group (for a full list of studied mRNA with mean fold changes and statistics see Supplementary Table A1).

### 3.6 Increased locomotor activity of aging α-synuclein-depleted mice

**Fig. 8:**
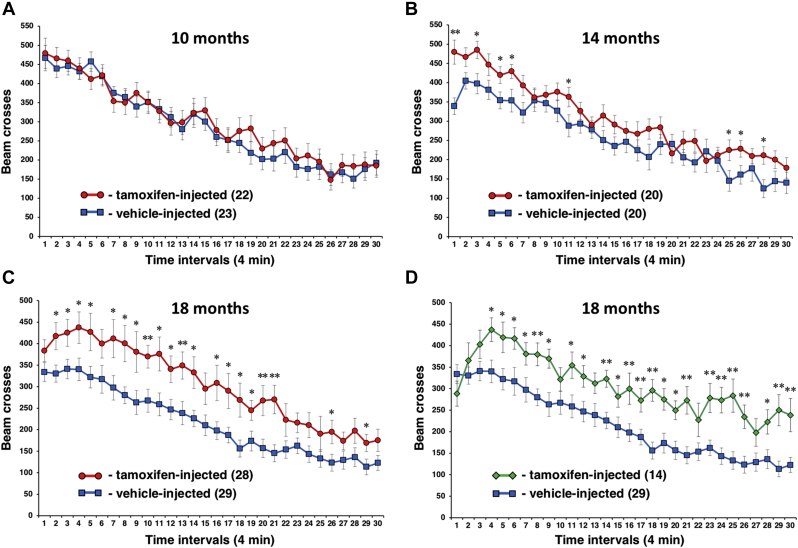
Analysis of animal locomotion in the activity camera. Groups of *Snca*^*flox/-*^ mice injected with tamoxifen or vehicle at the age of 6 months were tested at the age of 10 (A), 14 (B) and 18 (C) months and a group of *Snca*^*flox/-*^ mice injected with tamoxifen at the age of 12 months was tested at the age of 18 months (D). The number of animals in each group is shown in brackets. Graphs show mean ± SEM of the number of infrared beam crosses per 4-min interval. Two-way ANOVA revealed no significant difference in locomotion (beam breaks) between tamoxifen-injected and vehicle-injected groups tested at 10 months of age (F (1, 43) = 0.2120, *p* = 0.6475), but this difference was significant for 14 months (F (1, 38) = 4.231, *p* = 0.0466), 18 months (F (1, 55) = 8.212, *p* = 0.0059) and 18 months injected at 12 months (F (1, 41) = 12.18, *p* = 0.0012) groups. The presence of statistically significant difference for particular time interval identified by *post-hoc* Fisher test is also indicated (∗*p* <0.05, ∗∗*p* <0.01).

In the previous study, we demonstrated that constituent inactivation of genes coding for all three members of the synuclein family caused hyperactivity of young adult triple knockout mice (Anwar et al., 2011). Conditional inactivation of only an α-synuclein-encoding gene in adult animals triggered the gradual development of a similar phenotype. 10-month-old *Snca*^*flox/-*^ mice injected with tamoxifen at the age of 6 months showed the same locomotor activity in a nonanxiogenic activity camera as vehicle-injected mice (Fig. 8A), but 14-month-old mice displayed a hyperactive phenotype (Fig. 8B) that became profound in 18-month-old mice (Fig. 8C). Mice injected with tamoxifen at the age of 12 months developed a similar hyperactive phenotype at the age of 18 months (Fig. 8D).

## 4 Discussion

### 4.1 Depletion of α-synuclein in adult neurons

Tamoxifen-induced activation of pan-neuronally expressed Cre-ERT2 recombinase in adult (6-month-old), or aging (12-month-old) mice allowed us to effectively inactivate a floxed *Snca* gene in their neurons, which caused the reduction of a normally high level of α-synuclein in the dorsal striatum to a virtually undetectable level in the majority of treated animals. Assuming that changes in the nigrostriatal system of mice with complete germline inactivation of *Snca* gene (i.e., constituent α-synuclein knockout mice) are minimal because of an efficient compensation of α-synuclein function during a period of high plasticity of the developing nervous system, we expected to observe more profound changes in animals depleted of α-synuclein long after the end of this period. However, similar to previous observations made in constituent α-synuclein knockout mice, striatal contents of dopamine, its metabolites, TH, synaptophysin, as well as balance and coordination of mice, appeared unchanged at any of studied age points following inactivation of *Snca* gene at the age of 6 months. Moreover, neither striatal dopamine content nor balance and coordination were affected in 18-month-old mice following inactivation of *Snca* gene at the age of 12 months.

### 4.2 α-synuclein is not required for survival of mature dopaminergic neurons in the mouse SNpc

Previous studies demonstrated reduced complement of SNpc TH-positive neurons in two lines of mice constituently lacking α-synuclein (Al-Wandi et al., 2010, Connor-Robson et al., 2016, Garcia-Reitboeck et al., 2013, Robertson et al., 2004) and recently we made the same observation (manuscript in preparation) in a new mouse line with a constituent α-synuclein knockout produced by germline inactivation of *Snca* gene in *Snca*^*flox*^ mice (line *Snca*^*Δflox/Δflox*^; C57BL/6-Snca<tm1.1Vlb>/J; JAX Stock#025636) described in our previous publication (Ninkina et al., 2015). It has been shown that this deficit of neurons is established in the embryonic brain and is not progressive, suggesting the importance of α-synuclein for efficient genesis or survival of dopaminergic neurons or a certain specific population of these neurons only during a particular period of development (Garcia-Reitboeck et al., 2013). This implies that the depletion of α-synuclein in adult animal neurons should not cause their loss. Indeed, inactivation of *Snca* gene at the age of either 6 or 12 months did not lead to a reduction in the number of TH-positive neurons in the SNpc of 18-month-old mice. Therefore, similar to the majority of developing neurons, adult dopaminergic neurons of the SNpc are able to efficiently compensate for the absence of α-synuclein.

### 4.3 Late-onset depletion of α-synuclein affects dopamine catabolism in the dorsal striatum

Although an adult-onset depletion of α-synuclein does not affect the complement of dopaminergic neurons in the SNpc, it might still affect certain molecular processes in the neuronal compartment where this protein is normally most abundant and plays important roles in neurotransmission, namely in presynaptic terminals located in the dorsal striatum. Whereas α-synuclein depletion from the age of 6 months did not change the content of major dopamine metabolites in the dorsal striatum of aging mice, its depletion from the age of 12 months caused substantial decrease of both DOPAC and HVA. With the level of dopamine not affected, this caused a sharp decrease of the metabolites to dopamine ratio in these 18-month-old mice, in contrast to a slight increase of this ratio in 18-month-old mice with α-synuclein depletion from the age of 6 months. These observations suggest that in adult mice, dopaminergic neurons of SNpc are able to normalize dopamine turnover in their presynaptic terminals affected by the loss of α-synuclein, but in the aging nervous system this ability is compromised. It is feasible that in the dopaminergic presynaptic terminals in the dorsal striatum of mice depleted from α-synuclein later in their life dopamine catabolism is slowed down leading to accumulation of DOPAL and HVAldehyde, highly neurotoxic reactive aldehydes (Burke et al., 2003, Marchitti et al., 2007). Aldehyde dehydrogenases, particularly cytoplasmic ALDH1a1, are responsible for detoxication of DOPAL and HVAldehyde by their conversion to DOPAC and HVA, correspondingly. The role of these enzymes in the normal function of dopaminergic neurons (Anderson et al., 2011, Liu et al., 2014, McCaffery and Drager, 1994) and implication of their dysfunction in PD and other neurodegenerative diseases have been documented (Burke et al., 2008, Fitzmaurice et al., 2013, Galter et al., 2003, Wey et al., 2012). Although the experimental setup of our study did not allow for measuring levels of DOPAL and HVAldehyde, we have demonstrated that expression of mRNAs encoding enzymes involved in the production of aldehydes from dopamine was not affected in mice depleted of α-synuclein from the age of 12 months, but the expression of ALDH1a1 mRNA was reduced, and the level of this enzyme in the striatum followed the same trend. We appreciate that experimental evidence obtained so far is not conclusive enough to claim that the deficiency of ALDH1a1 is the reason for the reduced content of DA metabolites and that this reduction is linked with the accumulation of toxic aldehydes in the striatum of mice with late-onset depletion of alpha-synuclein. However, this is a possible scenario that deserves to be taken into consideration. Further studies will demonstrate if alterations of dopamine turnover indeed cause accumulation of toxic metabolites with potential effects on structure or/and function of dopaminergic synapses, but in the absence of obvious phenotype, one can suggest that any such changes would not be dramatic. However, it could not be excluded that in the aging brain affected by PD pathology, depletion of a functional pool of monomeric α-synuclein in presynaptic terminals could exacerbate malfunction of dopamine neurotransmission caused by the toxicity of oligomeric products of alpha-synuclein aggregation. Interestingly, it has been shown that DOPAL can induce aggregation of α-synuclein (Burke et al., 2008), and therefore, it is possible that a previously unrecognized vicious circle might develop in dopaminergic neurons after α-synuclein aggregation first triggered by seeding or an alternative mechanism.

### 4.4 No significant changes of neurotransmission-related mRNA expression in the midbrain of aging mice after a late-onset depletion of α-synuclein

Although no statistically significant differences in expression of neurotransmission-related mRNAs between α-synuclein-depleted and control midbrains have been detected by NanoString/nCounter approach, this comparative analysis revealed some trends that justify future detailed studies of particular mRNA and protein expression in more defined neuronal populations. Of particular interest is a slight increase in expression of mRNAs for two major dopamine receptors, D1 and D2 (Drd1 and Drd2), in tamoxifen injected animals, whereas mRNAs for dopamine (DAT, Slc6a3) and vesicular monoamine (VMAT2, Slc18a2) transporters were found predominantly downregulated in these animals. Such small but coordinated changes might reflect either consequence of the loss of α-synuclein function in presynaptic terminals of dopaminergic neurons of aging mice or functional compensation for this loss in the nigrostriatal system of these animals. It should be noted that expression was studied only in 18-month old mice, i.e. when natural aging might interfere with the efficacy of compensatory processes, and for mRNA encoding proteins involved in such processes, changes of expression could be more profound at earlier stages of adaptation to α-synuclein loss.

An example of age-related fading of compensation for the absence of α-synuclein can be illustrated by changes of mouse behavior observed in mice after adult-onset depletion of this protein. In a previous study, we revealed that the loss of all three synucleins leads to a significant increase of locomotor activity of young triple constituent knockout mice in a novel nonaxiogenic environment (Anwar et al., 2011). Here we demonstrated that mice depleted of α-synuclein from adulthood gradually develop similar hyperactive phenotype, indicating that certain mechanism, which may be linked to the presence of β-synuclein and γ-synuclein, initially compensate for the α-synuclein loss but their efficiency decline in the aging animals.

### 4.5 Is the reduction of α-synuclein level in adult neurons a safe long-term therapeutic approach?

Cutting the level of endogenous α-synuclein in PD patients has been suggested as a potential therapeutic approach to prevent the progression of the disease (Bae et al., 2012, Masliah et al., 2005, Masliah et al., 2011, Tran et al., 2014). Both RNA interference (Takahashi et al., 2015) and immunotherapeutic (reviewed in (Sardi et al., 2018, Wang et al., 2019)) approaches have been suggested, and clinical trials for some of them are already ongoing.

However, the results of some published studies raised uncertainty about the potential adverse effects of α-synuclein downregulation on the nigrostriatal system function. In two papers from the same laboratory authors attempted to halt α-synuclein expression in SNpc neurons of adult rats and green monkeys using an RNAi-mediated degradation of α-synuclein-encoding mRNA following unilateral injection of shRNA-expressing AAV5 virus particles in the SNpc (Collier et al., 2016, Gorbatyuk et al., 2010). Surprisingly, in these experiments striking neuronal loss of dopaminergic neurons in the SNpc, dramatic reduction of TH and dopamine content in the striatum and the case of rats, profound amphetamine-induced rotational asymmetry were observed in spite of only partial reduction of α-synuclein expression in SNpc neurons and scant decrease of its level in the striatum (Gorbatyuk et al., 2010). In another study, injection of shRNA-expressing AAV2 virus particles into the rat SNpc decreased the level of α-synuclein in the cell bodies of dopaminergic neurons by ~35%, but this did not cause any loss of SNpc neurons, or reduction of TH expression level, or movement deficit (Zharikov et al., 2015). Advert effects on the nigrostriatal system have not been observed in other studies that used in vivo delivery of naked (Lewis et al., 2008, McCormack et al., 2010) or exosome-packed siRNA against α-synuclein (Cooper et al., 2014). Possible explanations of these discrepancies have already been discussed (Zharikov et al., 2015).

Importantly, in contrast to all studies based on RNA interference, in our study, we achieved a complete cease of α-synuclein production in neurons and its depletion from the dorsal striatum. The permanent nature of this cease following loxP/Cre-driven inactivation of the gene allowed us to assess the long-term consequences of α-synuclein loss. We demonstrated that the nigrostriatal system is able to function properly for a long period of time after complete adult-onset depletion of α-synuclein from SNpc dopaminergic neurons and their presynaptic terminals.

## 5 Conclusions

Results of our study in a mouse model suggest that the reduction of the α-synuclein level does not per se causes long-term adverse effects on the functionality of adult neurons of SNpc, and therefore, can be considered as a safe option for therapeutic intervention. However, slight functional changes of the nigrostriatal system could be expected in the case of a late-onset depletion of α-synuclein, and it is not known if they might exacerbate pre-existing pathology in mid/late-stage PD patients. Therefore, to decrease the possibility of unwanted adverse effects, treatments aimed at reduction of α-synuclein level should be started at the early stages of PD.

## Disclosure statement

Declarations of interest: none.

## CRediT authorship contribution statement

**Natalia Ninkina:** Conceptualization, Methodology, Investigation, Validation, Visualization, Writing - original draft, Supervision, Funding acquisition. **Tatiana V. Tarasova:** Investigation, Visualization. **Kirill D. Chaprov:** Investigation, Visualization. **Andrei Yu Roman:** Investigation, Visualization. **Michail S. Kukharsky:** Investigation, Visualization. **Larisa G. Kolik:** Investigation, Visualization. **Ruslan Ovchinnikov:** Investigation, Visualization. **Aleksey A. Ustyugov:** Investigation, Visualization. **Andrey D. Durnev:** Resources, Formal analysis, Supervision. **Vladimir L. Buchman:** Conceptualization, Investigation, Formal analysis, Validation, Visualization, Writing - review & editing, Supervision, Funding acquisition, Project administration.

## Figures and Tables

**Supplementary Figure 1:**
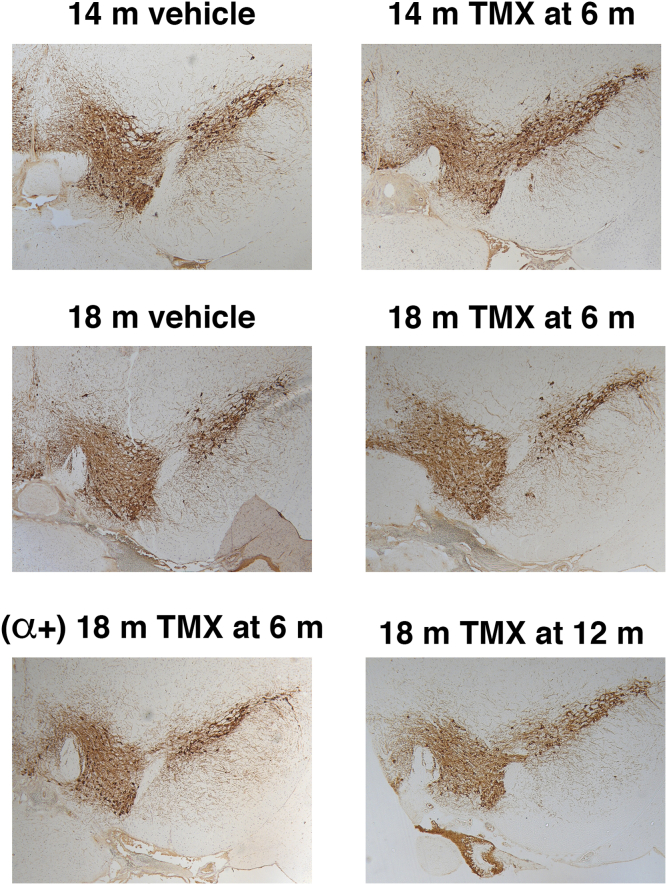
Representative images of brain sections immunostained with an antibody against TH.

## Appendix A Supplementary data

Supplementary Table A1 Expression levels of mRNAs in the midbrain of 18-month-old *Snca*^*flox/-*^ mice injected with tamoxifen or vehicle at the age of 12 months. Midbrains of four animals per group were used for nanoString/nCounter analysis of a bespoke codeset that included 106 mRNAs involved in synaptic transmission. Expression levels of 5 housekeeping RNA (β-actin, α-tubilin 1a chain, GAPDH, Hprt1, 60S ribosomal L4 protein, and 18S rRNA) were used for normalisation.

## Footnotes

1 It should be noted that it was not possible to assess accurately the number of neurons in the VTA of this smaller group of animals because following dissection their brains were cut sagittally and only one half of the brain was fixed for immunohistochemical analysis; this affected the integrity of VTA located close to the cut line, in contrast to more laterally located SNpc.
